# Fluoro-deoxi-glucose uptake and angiogenesis are independent biological features in lung metastases

**DOI:** 10.1038/sj.bjc.6600262

**Published:** 2002-05-06

**Authors:** G Veronesi, C Landoni, G Pelosi, M Picchio, A Sonzogni, M E Leon, P G Solli, F Leo, L Spaggiari, M Bellomi, F Fazio, U Pastorino

**Affiliations:** Department of Thoracic Surgery, European Institute of Oncology, Via Ripamonti 435, 20141 Milan, Italy; Nuclear Medicine Division, Ospedale San Raffaele, Via Olgettina 60, 20100, Milan, Italy; Department of Pathology, European Institute of Oncology, Via Ripamonti 435, 20141 Milan, Italy; Division of Statistic, European Institute of Oncology, Via Ripamonti 435, 20141 Milan, Italy; Division of Radiology, European Institute of Oncology, Via Ripamonti 435, 20141 Milan, Italy

**Keywords:** secondary lung cancer, angiogenesis, microvessel density, Fluoro-desoxi-glucose uptake, PET

## Abstract

Neoangiogenesis and enhanced glucose metabolism in neoplasms are likely to be activated by the same biochemical stimulus; hypoxia. A correlation between these two parameters has been postulated. The objective of this study was to evaluate the relationship between Fluoro-desoxi-glucose uptake at positron emission tomography scan and angiogenesis in lung metastasis. Fluoro-desoxi-glucose activity, expressed as a standard uptake value, and microvessel intratumoural density, were retrospectively calculated in a series of 43 lung metastasis resected in 19 patients. Primary sites were colorectal cancer in 16 metastases, sarcoma in eight, gynaecological in four and other sites in 15. The correlation between the two parameters was tested by logistic regression and multivariate analysis. Positron emission tomography scan was positive in 17 patients (sensitivity 89%). No correlation was observed between standard uptake value and microvessel intratumoural density in this series of lung metastasis. Positron emission tomography negative and positive nodules presented comparable value of microvessel intratumoural density (12.9 *vs* 11.3). Standard uptake value was significantly correlated with nodules size and was higher in colon cancer metastasis than in sarcoma ones. Microvessel intratumoural density was independent from nodule size but significantly higher in sarcoma than in colon cancer metastasis. The lack of correlation was confirmed by multivariate analysis after adjustment for tumour type and nodules size. The present study demonstrated that positron emission tomography scan is positive in a high proportion of patients regardless of microvessel density. Glucose uptake and angiogenesis appear to be independent biological features in lung metastasis. This observation may have implications for future antiangiogenic therapies.

*British Journal of Cancer* (2002) **86**, 1391–1395. DOI: 10.1038/sj/bjc/6600262 www.bjcancer.com

© 2002 Cancer Research UK

## 

The increasing use of positron emission tomography (PET) scan in management of different neoplastic diseases has brought great improvement in the diagnosis and staging of cancer patients particularly in relation to the preoperative selection of surgical candidates (Delbeke, 1999; Saunders *et al*, 1999).

Besides the staging implications of this new instrument, particular attention has recently been focused on the possible correlation between neoplasms glucose uptake and other biological parameters predictive of tumoural aggressiveness. Some investigators have already observed the capacity of tumoural glucose uptake, expressed by Standard Uptake Value (SUV), to predict tumour metastatic power and worse prognosis in lung cancer (Younes *et al*, 1997; Ahuja *et al*, 1998; Vansteenkiste *et al*, 1999) and other neoplasms (Oshida *et al*, 1981; Nakata *et al*, 1997; Pasquali *et al*, 1998; Benard *et al*, 1999). Similarly a large number of studies have documented a relationship between the angiogenic profile of a tumour (IMD) and its aggressiveness in NSCLC and other experimental and human models such as colonic, breast, gastric, renal and lung carcinomas (Harpole *et al*, 1996; Apolinario *et al*, 1997; Weidner, 1998; Breast Cancer Progression Working Party, 2000). In conclusion, even if these data are not confirmed for soft tissue sarcoma (Tomlinson *et al*, 1999), both SUV and IMD seem to represent unfavourable prognostic factors in epiteliar tumours, but their correlation has never been investigated.

Changes in the rate of glucose uptake and over-expression of glucose transporters in cancer cells are likely to be associated with adaptation to hypoxia in rapidly growing tumours (Kuiper *et al*, 1998), partly due to their increased dependency on glycolysis as energy source (Vauperl *et al*, 1989). Otherwise hypoxia, which is the result of a lack of perfusion and vascular compression by the tumour, may also contribute to the enhanced expression of specific angiogenic peptides and their receptors (Vauperl *et al*, 1989; Merral *et al*, 1993; Kuiper *et al*, 1998). Therefore it can be postulated that both angiogenesis and glucose metabolisms are activated by the same biochemical stimulus: hypoxia.

At present, the histological microvessel density technique is the gold standard (Fox, 1997) to characterise the degree of angiogenesis (Vermeulen, 1996), but a non invasive, objective techniques for preoperative assessment of tumoural vascularity, including glucose metabolisms at PET scan, are under investigation (Fanelli *et al*, 1999). To identify the type of relationship between glucose metabolism and neoangiogenesis may be of interest not only in the field of pulmonary metastases, but also in other neoplasms in order to assess angiogenesis, define prognosis and predict response to anti-angiogenic therapies (Ziche *et al*, 1999).

## PATIENTS AND METHODS

Between September 1998 and December 1999, 19 patients with lung metastases from different hystotypes underwent preoperatively PET scan. At the time of surgery, a total of 43 nodules were resected and proved to be metastatic. These cases represent 23% of lung metastasectomies performed in the same period. Primary sites were colorectal cancer in 16 nodules (10 patients), sarcoma in eight nodules (four patients), gynaecological in four nodules (two patients), other site in 15 nodules (three patients). Analysis of angiogenesis included 12 additional nodules (10 sarcoma and two colon) from three patients of the same population but resected before PET scan examination. Table 1

**Table 1:**
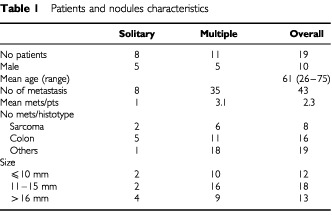
Patients and nodules characteristics

describes the patients and metastasis characteristics included in the analysis.

Images were acquired with a GE Advance PET scanner (General Electric Medical Systems, Milwaukee, US, axial field of view of 15 cm and resolution of approximately 5 mm). One hour after injection of approximately 370 MbQ of [18F]FDG, each patient was positioned supine on the tomographic bed with arms over the head and a whole-body emission scan (5 min per bed position) was started covering a field of view from neck to pelvis. Transmission scan (3 min per bed position) was then performed on thorax region to measure attenuation. Raw data were corrected for measured attenuation using segmented transmission data (ref. Bettinardi) and then reconstructed in transaxial images using an iterative algorithm with 16 subset and order 4. Parametric SUV transaxial images were obtained normalising each pixel for injected dose and body weight as follows: SUV=(pixel-by-pixel activity in Bq/cc)/(injected dose in MBq/body weight in kg). For the determination of the SUV, regions of interest (ROIs) were manually drawn on transaxial images around the focal FDG-uptake area. No correction for partial-volume effect was performed. Maximum value of mean SUV of different slices of each nodule was considered for statistical analysis.

Positive nodules at PET scan were identified within the lung during surgical resection to perform nodule specific correlation between standard uptake value (SUV) and histological microvessel density (IMD).

Surgical specimens were immunostained by a monoclonal antibody anti CD34 JC-70. Intratumoural capillaries and small venules were identified and counted by light microscopy using Chalkley counting, performed in three representative intratumoural and peritumoural areas of each slide in a ×200 field with a lens of 0.86 mm diameter. The sum of Chalkley counting was considered for statistical analysis. All the vessels counted determinations were performed by the same pathologist blinded from SUV data.

### Statistical analysis

Analysis was conducted treating nodules as independent units of observation because within-subject correlation on multiple nodules was very low (Proc Mixed procedure in SAS, Cary, NC, USA). Microvessel density (IMD), as sum of Chalkley counting, and maximum of mean standard uptake values (SUV) were used as continuous variables. In addition, SUV values were regrouped into negative (SUV=0) or positive (SUV>0) values to assess the sensitivity of PET. Chi-square test and Fisher Exact Extension test were used to compare the distribution of values across categorical variables. Analysis of covariance was used to generate least square SUV means adjusted by nodule size. Pearson product-moment and Spearman rank correlations were calculated to assess the association between angiogenesis and FDG-uptake variables, depending on compliance with the normality assumption. In addition, multiple linear regression was used to adjust for nodule size when assessing the linear association between glucose metabolism and angiogenesis. *P*-values were derived from two-sided tests. A statistical significant difference was established if the associated *P*-value was >0.05.

## RESULTS

PET scan was positive in 26 out of 43 metastases. Mean standard uptake value (SUV) was 3.9±2.5 (median 3.6) with a range from 1.2 to 9.5 (Table 2

**Table 2:**
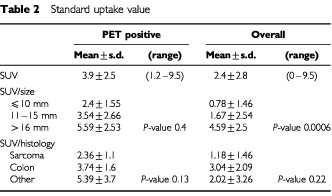
Standard uptake value

). SUV increased with increasing nodule size (*P*-value=0.0006), but no significant correlation was observed between SUV and nodule size when excluding PET negative nodules (*P*-value=0.4). Standard uptake value was higher in colon metastases than in sarcoma ones (3.04±2.09 *vs* 1.18±1.46), even though statistical significance was not reached (*P*=0.22).

As shown in Table 3

**Table 3:**
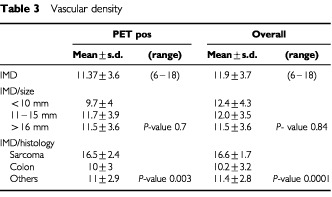
Vascular density

, mean intratumoural microvessel density was 11.9±3.7 (median 12), IMD was independent from nodule size (*P*-value=0.89) being significantly higher in sarcomas than in colon cancer (mean microvascular density: 16.6±1.7 *vs* 10.2±3.2, respectively; *P*<0.0001). In summary whilst colon cancer metastases showed high glucose metabolism and low vascular density, sarcoma metastases had low activity at PET and high vascular density.

No correlation was shown between glucose metabolism activity and microvessel density (Table 4

**Table 4:**
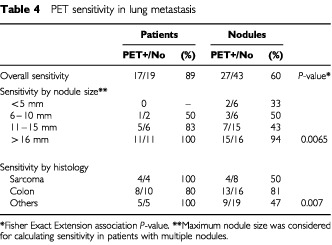
PET sensitivity in lung metastasis

), even after having adjusted for nodules size. Mean microvessel density in PET negative metastases was comparable with that found in PET positive nodules (12.9±3.9 *vs* 11.3±3.5 no significant difference). Comparison of the two variables by categorical versions of SUV did not reveal any difference (*P*=0.68). The lack of a significant correlation was evident even when excluding PET negative nodules from the analysis or after adjusting SUV data for nodule size, to exclude influence of dimension on SUV data. When stratifying nodules by their size, the correlation between microvessel density and SUV showed a borderline *P*-value only in nodules under 1 cm: −0.625 (*P*=0.07). Similarly, assessment of the correlation between IMD and FDG–SUV after having stratified nodules by histology did not result in significant differences.

Diagnostic value of PET scan was summarised in Table 4. Specificity was not evaluable since only malignant lesions were analysed in the present study. Overall sensitivity was 60% for nodules and 89% for patients. Only 33% of nodules smaller than 5 mm were PET positive, but sensitivity increased to 55% for nodules between 5 and 10 mm and 94% for nodules greater than 15 mm (*P*-value=0.0065). Sensitivity was higher for colon cancer metastases than for sarcoma nodules (81% *vs* 50%) but size factor clearly influenced these results, in fact sarcoma metastasis were significantly smaller than those from other types (12±8.7 mm *vs* 21.2±13.2; *P*=0.005). In sarcoma metastatic, vascular density tended to be higher when multiple nodules were present per patient (not significant): mean IMD was 15.5±3.5 in patients with one nodule only and 21.5±9.2 in patients with five nodules (two patients).

## DISCUSSION

In recent years a number of studies have demonstrated that the ability of a tumour to induce proliferation of new blood vessels has a profound effect on tumour growth, metastasis and prognosis (Harpole *et al*, 1996; Apolinario *et al*, 1997; Weidner, 1998; Breast Cancer Progression Working Party, 2000). Inhibition of angiogenesis and vascular targeting have become promising new anti-cancer strategies. As pointed out by Aurerbach *et al* (1991) ‘perhaps the most consistent limitation of antiangiogenic research is the availability of a simple, reliable, reproducible, quantitative assay of the angiogenic response’. Although quantitative assessment of tumour vascularization would be of major importance as a prognostic factor and for the choice of therapeutic strategies, for example as a way to select patients for clinical studies with antiangiogenic agents, the optimal method for assessing it has not been developed yet. At present, the most widely used method is the assessment of intratumoural microvessel density through immunohistochemical analysis with specific markers for endothelial cells (Vermeulen *et al*, 1996). To overcome the labourious nature of this method, the problem of the observer variation and reproducibility and invasiveness of the procedure, some investigators have started to explore non-invasive, *in vivo*, imaging-functional techniques to assess tumour vascularity, such as doppler sonography (Ferrara *et al*, 2000), dynamic contrast enhanced magnetic resonance imaging (Hawignorst *et al*, 1998), functional CT technique (Miles *et al*, 2000) and radionuclide imaging (Blankenberg *et al*, 2000). Even PET scan with FDG has been considered as a way to measure blood flow by assessment of glucose metabolism enhancement, based on the assumption that tumour influx of the glucose analogue, and therefore its uptake, should be proportional to blood flow and therefore to tumoural vascularity (Vermeulen *et al*, 1996), but no studies have so far investigated this subject. We postulated the existence of a relationship between neoangiogenesis and enhanced glucose metabolism, basing our hypothesis on the biological evidence that both neoangiogenesis and glucose metabolism were activated by the same stimulus: hypoxia. In addition, the clinical observation that these two variables may represent poor prognostic factors, have been suggested by some authors as an indirect factor of a potential link between the two variables.

Lung metastasis represent a favourable model to study neoangiogenesis and FDG–SUV, for the small size of nodules, the easily measurable diameter, their regular margins and their position within lung parenchima, faraway from major vessels, possible source of interference with SUV and IMD data. In spite of these advantages the main limit of this model is represented by the heterogeneity of tumour types.

In contrast with common sense expectation, the main finding of the current study was the lack of correlation between vascularity and glucose metabolism.

To better understand this unexpected finding we analysed the subgroups of different histotypes to verify if heterogeneity of primary tumour could influence the result. Subgroup analysis led to similar results. In addition, to eliminate the influence of nodule size on SUV–IMD correlation we adjusted SUV by nodule size, but no significant result emerged.

The two main histotypes, colon and sarcoma metastasis, have suggested an opposite biological behaviour: whilst sarcomas showed high vascular density and low glucose activity, colon cancer nodules presented low vascular density and high glucose activity. The observed higher IMD in sarcoma than in carcinoma should be taken with caution in spite of the *P*-value because of the limited number of sarcoma cases and the method of counting metastases from the same patient as separate observations.

These data suggest that neoangiogenesis and glucose metabolism are independent factors and that PET scan is unable to predict tumour angiogenic patterns in lung metastases. But further investigations about PET and angiogenesis are necessary for primary lung neoplasms.

Although this conclusion would deny a specific capacity of PET scan to predict response to antiangiogenic therapy, it does not exclude the potential role of this technique in monitoring a tumours response to standard anticancer therapy or to identify recurrent or persistent disease after chemotherapy or radiotherapy as suggested by some authors (Akhurst and Larson, 1999). The functional property of PET imaging is its ability to evaluate changes of tumour metabolism, beyond and maybe before anatomical modifications.

A large number of studies have defined the role of angiogenesis in primary tumour growth and progression (Horak *et al*, 1992; Weidner and Folkman, 1996) suggesting a relationship between size of tumours and neoangiogenesis. In contrast with these findings, our results in lung metastasis showed that microvessel density was independent from nodule size. IMD tended to increase with number of lung metastatic deposits in case of sarcoma metastasis, suggesting that aggressiveness of the disease may not be related to lung lesions size but to their number. These hypothesis would be in accordance with previous observations that identified number of metastases as an independent negative prognostic factor (Pastorino *et al*, 1998). From a pathological point of view, the lower vascular density of colon cancer metastasis in comparison with sarcoma metastasis may be related to the intense central necrosis observed in colon cancer metastasis but the biological and prognostic meaning of these observations are not clear and only the follow up study of this population may clarify this aspect in the future.

Contrasting results emerge from the literature on the relationship between SUV and malignant nodule size. Brown *et al* (1999) observed a correlation between FDG uptake and tumour diameter in 23 lung cancers while Duhaylongsod *et al* (1995) didn't find any correlation in 36 primary lung tumours. In the present study FDG uptake measured by SUV was significantly correlated with nodule size when including both PET positive and negative metastases, but no significance was observed when only PET positive metastases were considered for analysis (*P*-value=0.4). This information should be taken into account when SUV is evaluated in clinical studies, to avoid FDG activity being confounded by nodule size. In addition it may contribute useful information when FDG metabolism is investigated as a diagnostic and prognostic tool.

Although the high proportion of PET negative nodules in this study does not compare favourably with previous reports, PET was able to identify lung malignancy in 17 of the 19 patients with a sensitivity of 89%. If we consider the number of nodules, PET scan showed an overall sensitivity of only 60%, proportional to tumour diameter: Thirty-three per cent for nodules smaller than 5 mm, 55% for nodules between 5 and 10 mm and 94% for nodules greater than 15 mm (*P*-value=0.0065). Sensitivity was higher for colon cancer than sarcoma metastasis (81 *vs* 50%). But this data is influenced by nodule size factor (being sarcoma metastasis significantly smaller than colon cancer). The reported data on PET scan sensitivity represent preliminary results but the clinical role of PET in lung metastases will be further studied and described in a separate analysis.

In conclusion the observed lack of correlation between angiogenic activity and glucose uptake in lung metastasis represents a novel finding in cancer biology. PET scan seems unable to predict angiogenic patterns of tumour in lung metastases but further investigations are necessary to confirm these data on primary neoplasms.

## Note added in proof

Fluoro-desoxi-glucose activity, expressed as a standard uptake value, and angiogenesis, expressed by microvessel intratumoural density were retrospectively calculated in a series of 43 resected lung metastasis. Uni- or multivariate analysis showed no correlation between the two parameters. This observation may have implications for future antiangiogenic therapies.
